# Stereotactic radiotherapy using the CyberKnife is effective for local control of bone metastases from differentiated thyroid cancer

**DOI:** 10.1093/jrr/rrz056

**Published:** 2019-08-19

**Authors:** Takayuki Ishigaki, Takashi Uruno, Kiminori Sugino, Chie Masaki, Junko Akaishi, Kiyomi Y Hames, Akifumi Suzuki, Chisato Tomoda, Kenichi Matsuzu, Keiko Ohkuwa, Wataru Kitagawa, Mitsuji Nagahama, Shinichiro Miyazaki, Koichi Ito

**Affiliations:** 1 Department of Surgery, Ito Hospital, 4-3-6 Jingumae, Shibuya-ku, Tokyo 150-8308, Japan; 2 Department of Breast and Endocrine Surgery, The Jikei University School of Medicine, 3-25-8, Nishi-shinbashi, Minato-ku, Tokyo 105-8461, Japan; 3 Department of Radiation Oncology, Shinyurigaoka General Hospital, 255, Furusawatsuko, Asou-ku, Kawasaki-shi, Kanagawa 215-0026, Japan

**Keywords:** stereotactic radiotherapy, CyberKnife, differentiated thyroid cancer, bone metastasis

## Abstract

Differentiated thyroid cancer (DTC) is associated with a good long-term prognosis, but bone metastases can adversely affect patients’ quality of life and survival. Stereotactic radiotherapy (SRT) can deliver high-dose irradiation to target lesions and it has been reported to be useful for various cancers. However, few studies have examined the efficacy of SRT for thyroid cancer. In the present study, the aim was to investigate the efficacy of SRT using the CyberKnife for bone metastases from DTC. From September 2013 to April 2018, SRT with the CyberKnife system was used to treat 60 bone metastases from DTC in 13 patients. The patients’ medical records were retrospectively reviewed to obtain information about the adverse events associated with SRT. Of the 60 lesions, 40 could be evaluated by follow-up CT for therapeutic effectiveness, and the RECIST criteria were used to assess the response. The cancers were papillary cancer in 3 patients, follicular cancer in 9 and poorly differentiated cancer in 1. SRT was delivered in 1–10 fractions, with a median dose of 27 Gy (range, 8–48 Gy). Adverse events were infrequent and mild. The median follow-up of the 40 lesions was 11 (range, 2–56) months. The responses were partial response in 2 lesions, stable disease in 37 lesions and progressive disease in 1 lesion, with a 1-year local control rate of 97.1%. The present study showed that SRT using the CyberKnife system was a feasible and effective treatment for bone metastases of DTC.

## INTRODUCTION

Differentiated thyroid cancer (DTC) is associated with good long-term cause-specific survival (CSS). However, distant metastases including bone metastases can adversely affect patients’ quality of life and CSS [1]. The CSS from diagnosis of initial bone metastasis was 36% at 5 years and 10% at 10 years in one study [2]. Radioactive iodine (RAI) therapy has been found to be associated with improved survival of patients with metastases from DTC and should be given for iodine-avid bone metastases, but RAI is rarely curative [3]. For RAI-refractory and progressive bone metastases from DTC, local therapies are required. For patients diagnosed with bone metastases, several treatment options exist for the management of local disease, including surgical intervention, radiotherapy or a combination of these therapies [1]. The role of conventional external beam radiotherapy (EBRT) for bone metastasis treatment is well established, and the goals of EBRT are to remove the cause of pain, to improve paralysis or to prevent pathologic fractures or spinal cord compression [4]. However, EBRT has been found in trials to result in higher rates of local progression and pain relapse with long-term follow-up [5, 6]. One study reported local tumor progression in as many as 70% of patients at 1 year as evaluated by radiographic findings after EBRT [5]. On the other hand, compared with conventional EBRT, stereotactic radiotherapy (SRT) demonstrated higher efficacy in tumor control by enabling delivery of high-dose irradiation to lesions [3, 7]. Recently, a single Japanese institution reported the efficacy of SRT for spine metastases from various cancers. The 1-year local control rate was more than 80% even in radioresistant tumors such as renal cell carcinoma, sarcoma or thyroid cancer, but the details of characteristics, including the histology of thyroid cancer, were unknown [8]. Few studies have yet addressed the efficacy of SRT for thyroid cancer including bone metastases, and there has been no study of SRT for non-spine bone metastases from thyroid cancer. In a recent report, we showed the usefulness of SRT for locoregional recurrence of DTC, with a local control rate (LCR) of 84.6% at 3 years [7]. The aim of the present study was to examine the feasibility and efficacy of SRT using the CyberKnife (Accuray, Sunnyvale, CA, USA) as a salvage treatment for bone metastases from DTC, including spine and non-spine lesions.

## MATERIALS AND METHODS

### Patients’ characteristics and previous treatments

From September 2013 to April 2018, 13 (10 female, 3 male) patients with a median age of 69 years (range, 42–87 years) underwent SRT using the CyberKnife for bone metastases from DTC. These patients’ medical records were reviewed retrospectively. The cancers were papillary thyroid cancer (PTC) in 3, follicular thyroid cancer (FTC) in 9 and poorly differentiated cancer in 1. At the time of SRT, synchronous distant metastases other than bone metastases were present in 6 patients. All patients underwent neck surgery more than once and 10 patients received RAI therapy as postoperative ablation or treatment for distant metastases including bone metastases. Two patients underwent surgery for bone metastases before SRT. One patient underwent corpectomy and posterior spinal fusion for lumbar spine metastasis causing pain in the lower limb, but SRT was performed for relapse of pain in the lower limb 29 months after surgery. Another patient underwent posterior decompression and fixation for cervical spine metastasis to prevent fracture, followed by SRT for the same lesion. Two patients had received EBRT to the bone metastatic (BM) lesion before SRT. In one patient with metastases to lung, brain and pancreas, tyrosine kinase inhibitor (TKI) treatment was given before SRT. Table 1 summarizes the patients’ characteristics and previous treatments.

**Table 1:** Characteristics of the 13 patients and 60 lesions

Patients (*n* = 13)	
Median age (range)	69 years (42–87)
Male/female	3/10
Histology	
Papillary cancer	3
Follicular cancer	9
Poorly differentiated cancer	1
TNM stage^*^ at the time of initial surgery	
II	3
III	3
IVA	2
IVC	4
Unknown	1
Distant metastases other than bone metastases at the time of SRT	
Nothing	7
Lung	2
Lung and brain	2
Liver	1
Lung, brain and pancreas	1
Median duration from initial surgery to SRT (range)	52 months (1–380)
Previous treatment	
RAI therapy yes/no	10/3
(range of cumulative dose)	(1110–18 870 MBq)
Surgery yes/no	2/11
EBRT yes/no	2/11
TKI treatment yes/no	1/12
Number of treated lesions per patient	
1	4
2	3
3	1
4	2
9	1
29	1
Lesions (*n* = 60)	
Site of lesion	
Skull	2
Cervical spine	7
Thoracic spine	11
Lumbar spine	10
Ribs	10
Scapula	5
Pelvis	11
Others^**^	4
Median size of lesions (range)	21 mm (5.5–70)
Symptoms caused by bone metastasis yes/no	7/53
Median number of fractions (range)	3 fx (1–10)
Median dose of SRT (range)	27 Gy (8–48)

^*^TNM Classification of Malignant Tumours 7th ed, SRT=stereotactic radiotherapy, RAI=radioactive iodine, EBRT=external beam radiotherapy, TKI=tyrosine kinase inhibitor.

^**^Others include hyoid, clavicles and sternum.

### Profiles of target lesions and the details of SRT

A total of 60 BM lesions in 13 patients were treated by SRT using the CyberKnife system. The median diameter of the metastatic lesions was 21 mm (range, 5.5–70 mm). Seven of 60 lesions caused pain, numbness of the limb or both. SRT was delivered in 1–10 fractions, with a median dose of 27 Gy (Table 1).

### Radiotherapy

**Fig. 1:**
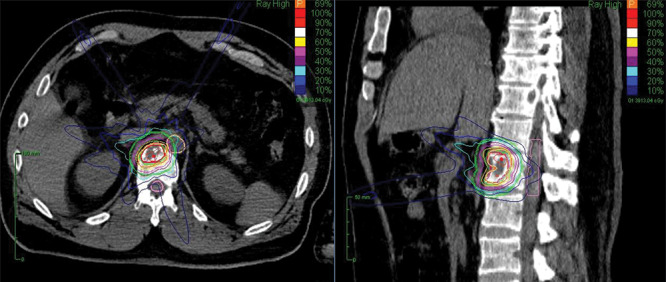
The left and right panels are axial and sagittal CT images, respectively, with contouring for planning SRT.

SRT using the CyberKnife was given in institutions located in Tokyo. Irradiation planning, including the fraction and dosage, was performed by experienced radiologists who considered lesion size and location. The gross tumor volume (GTV) was defined as visible tumor on CT and positron-emission tomography CT fusion (PET-CT), with images merged for target definition. GTV was considered the same as clinical target volume (CTV). The planning target volume (PTV) included the CTV and a margin of 1.2–2.0 mm. The prescribed dose to the target volume and the number of fractions were 21–27 Gy and three fractions, respectively, though they were adjusted depending on target tumor size and with or without previous irradiation. Furthermore, SRT was planned while considering the dose constraints of the organs at risk, such as the spinal cord, the aorta and the large intestine. A representative SRT plan is detailed in Fig. 1.

### Follow-up and evaluation

Information about adverse events and associated symptoms related to SRT was collected for 60 BM lesions in 13 patients. Assessment of adverse events was performed using Common Terminology Criteria for Adverse Events Version 4.0 [9]. Of the 60 BM lesions, 40 were measurable and assessable and could be evaluated by follow-up CT for therapeutic effectiveness. The RECIST criteria were used to assess response [10], with all measurements performed on CT scans. Metastases that had a long axis length ≥10 mm were considered measurable and assessable as target lesions. Response was assessed using the following definitions: complete response (CR), disappearance of the target lesions; partial response (PR), ≥30% decrease in diameter of the target lesion; progressive disease (PD), ≥20% increase in diameter of the target lesion and an absolute increase of ≥5 mm; and stable disease (SD), neither sufficient shrinkage to qualify for PR nor sufficient increase to qualify for PD. Local control was defined as CR, PR and SD on CT. CSS was defined as the time from the date of SRT to the date of death from DTC.

### Statistical analysis

The LCR and CSS were estimated using the Kaplan-Meier method. A statistical software package (JMP 11.0, SAS Institute, Inc., Cary, NC, USA) was used for statistical analyses.

This study was approved by the institutional review board at Ito Hospital (number 248).

## RESULTS

### Imaging outcomes

**Fig. 2:**
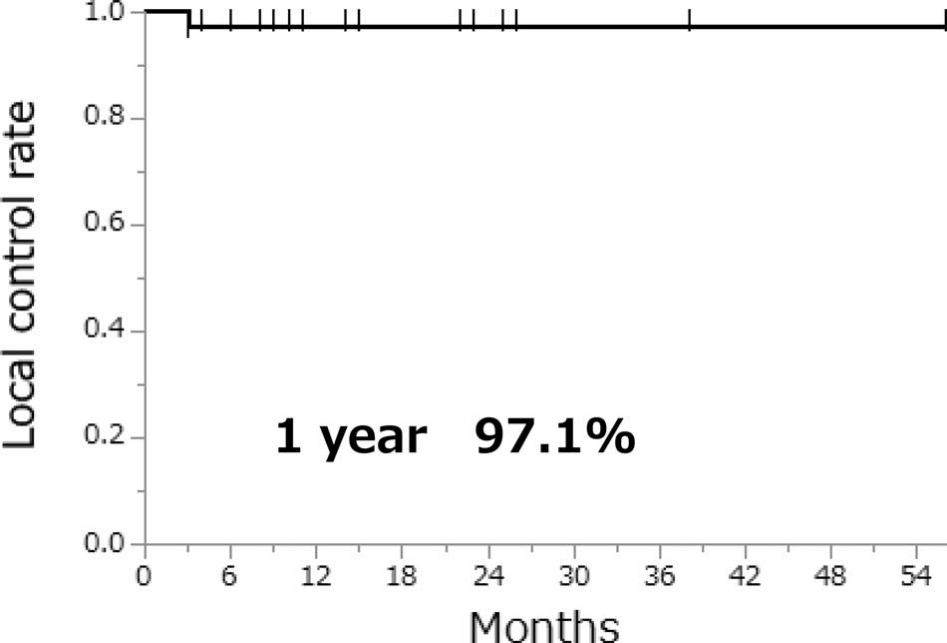
Local control rate of 40 lesions after SRT.

**Fig. 3:**
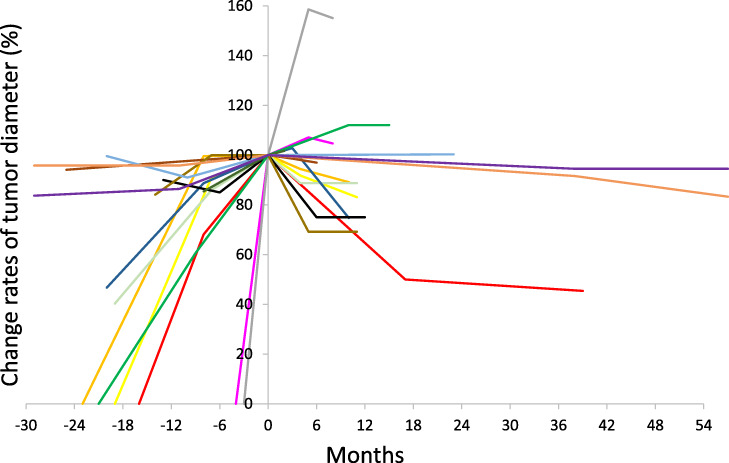
Change rates of tumor diameter before and after SRT in 15 lesions of 9 patients. The tumor diameter at SRT was set to 100%. Most irradiated tumors show tendencies to shrink or decrease in growth rate after SRT.

The 40 lesions could be evaluated by follow-up CT for therapeutic effectiveness, with a median follow-up period of 11 (range, 2–56) months. Responses were PR in 2 lesions, SD in 37 lesions and PD in 1 lesion (Table 2). Fig. 2 shows a 1-year LCR of 97.1% in 40 lesions. Fig. 3 shows the rates of change in tumor diameter before and after SRT in 15 lesions of 9 patients. The other 25 lesions were excluded because the change in tumor diameter before SRT was unknown. The tumor diameter at SRT was set to 100%. Most irradiated tumors showed tendencies to shrink or decrease in growth rate after SRT.

**Table 2:** Treatment outcomes of SRT for 40 lesions in 11 patients

Follow-up period	
Median (range)		11 months (2–56)
Response (*n* = 40)		*n* (%)
CR		0 (0)
PR		2 (5)
SD		37 (92.5)
PD		1 (2.5)

CR=complete response, PR=partial response, SD=stable disease, PD=progressive disease.

### Clinical outcomes

Clinical outcomes were evaluated for 60 BM lesions in 13 patients treated by SRT, with a median follow-up period of 11 (range, 1–58) months. Table 3 shows the adverse events that were associated with SRT. Adverse events were mild and of limited duration (within 2 months).

**Table 3:** Adverse events associated with SRT for 60 lesions in 13 patients

	Irradiated site	Grade	*n*
Esophagitis	Thoracic spine	≤2	1
Vomiting	Cervical spine	1	1
Nausea	Rib	1	1
Dermatitis	Thoracic spine	1	1

After SRT, the symptoms were improved in 6 (85.7%) of 7 lesions causing pain or numbness, but the symptoms were not improved in 1 sacral lesion with pain of the buttocks and numbness of the left lower limb. In 1 lumbar lesion, second SRT was required for relapse of low back pain 32 months after the first SRT, while no pain progression was observed in the other 5 lesions in which pain was relieved after SRT, for a median follow-up period of 8.5 (range, 1–39) months. During the clinical follow-up period, no new symptoms occurred in 53 lesions with no symptoms at SRT.

**Fig. 4:**
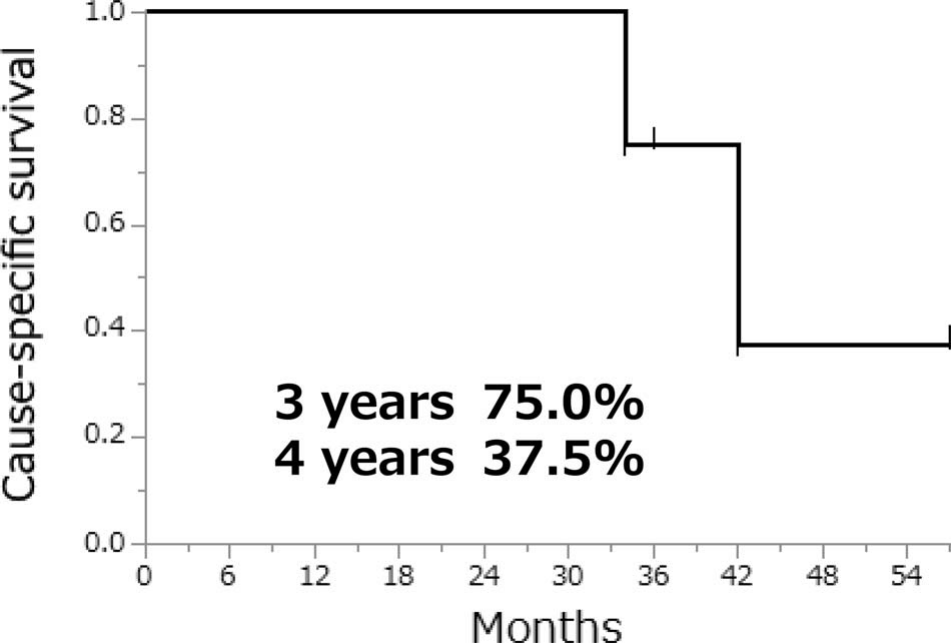
Cause-specific survival of 13 patients after SRT.

Fig. 4 shows the CSS of 13 patients after SRT. The 3-year CSS was 75.0% and the 4-year CSS was 37.5%. Two patients died of DTC, one due to asphyxia caused by large local recurrence in the neck and the other due to renal dysfunction because the patient’s general condition declined with systemic metastases. There were no deaths due to target lesion progression after SRT.

## DISCUSSION

Bone is the second most common site of distant metastases from thyroid cancer after the lungs. Patients with distant metastases, particularly to bone, have a marked decrease in survival [2]. In addition, progression of bone metastases, with pain, numbness and fracture, is directly linked to decreased quality of life. Therefore, in the treatment of thyroid cancer, as well as other cancers, management of bone metastases is very important and RAI therapy and TKI treatment specific to thyroid cancer are treatment options.

RAI therapy should be given for iodine-avid bone metastases, although it is rarely curative [3]. In one retrospective study that summarized 109 patients with bone metastases from DTC, multivariate analysis showed that the cumulative dose of RAI therapy was associated with improved survival [11]. In a multicenter study of 143 patients with bone metastases from DTC, both bone surgery and EBRT did not appear to significantly affect the clinical outcomes of bone metastases, whereas RAI therapy was shown to have favorable effects on patient survival [12]. On the other hand, RAI therapy has limitations in treatment. Another study of 444 patients with distant metastases from DTC reported that RAI therapy was highly effective in patients with ^131^I uptake who were <40 years of age and had small metastases, whereas RAI therapy should be abandoned and other treatment modalities should be used in patients who achieved no response or had no ^131^I uptake. In that study, 27.4% of the patients with bone metastases had no ^131^I uptake [13]. In the present study, most of the 10 patients who received RAI therapy eventually had become RAI-refractory at the time of SRT. The other 3 patients did not undergo RAI therapy, and other treatments including SRT were prioritized due to a cervical spine lesion with possible fracture, large skull metastasis pressing the brain or poor general condition.

For RAI-refractory bone metastases, surgical palliation and/or other local therapies such as EBRT should be considered when available if symptomatic or if asymptomatic in weight-bearing sites [14]. The objective of EBRT is to alleviate pain and neurological complications of bone lesions. Data on the efficacy of EBRT in the management of thyroid cancer and bone metastases are lacking, but it is thought that 70% of patients experience pain relief with palliative EBRT [15, 16]. Although very little data were found with respect to toxicity associated with EBRT for bone metastases, short-term reversible toxicity such as fatigue, mucositis or bowel irritation, and more feared complications such as radiation myelopathy, were reported [4]. Re-irradiation with EBRT may be feasible and effective, though retreatment to sites including radiation-sensitive critical structures may prove risky [17, 18]. Therefore, other treatment modalities should be selected in some cases.

The CyberKnife is an SRT device with image-guidance that consists of a robot arm, a linear accelerator and a target tracking system. With this system, one can irradiate a target with less damage to proximal organs by moving the robot arm, which has a wide range of motion. With SRT, precise delivery of high-dose radiation to the target can be achieved using a small number of fractions [7]. SRT compared with conventional 3D conformal radiotherapy demonstrated a higher efficacy in tumor control of bone metastases and in limiting radiation to the spinal cord, especially in patients who needed repeat irradiation [3]. Retrospective studies of SRT for bone metastases from different cancers demonstrated high local control rates of approximately 80–90%, one of which identified metastases from colorectal cancer and radiation history as independent predictors of lower local and pain control rates [4, 8, 19]. In phase 1/2 trials, patients with spine metastases from DTC were prospectively enrolled. In 27 spine lesions of 23 patients, LCR was 88% at 2 years and 79% at 3 years, with a median follow-up period of 28.9 months, and there were no reported events of myelopathy, radiculopathy or other grade 3–5 toxicity following SRT. Of eight patients who underwent SRT as re-irradiation for progressive disease following EBRT, only one showed evidence of further progression at 3 years [1].

In the present study, no local progression was observed, except for one of 40 bone metastases followed by CT scan after SRT, and adverse events associated with SRT for 60 bone metastases were not frequent and mild. In addition, there was neither progression nor adverse events in 2 lesions, including the lumbar spine and sacrum, treated by SRT as re-irradiation following EBRT. In Fig. 3, most tumors irradiated by SRT showed tendencies to shrink or decrease in growth rate, which may suggest the effect of SRT to suppress the growth of bone metastases. In addition, the treatment response to bone metastases is generally difficult to evaluate on CT scan images. In the present study, CR, PR and SD lesions were considered locally controlled. The only case of PD was a 69-year-old woman who underwent SRT (27 Gy/3 fx) for a 14.5-mm-diameter, lower thoracic spine metastasis from PTC, and it increased to 23 mm on CT scan 4 months after SRT. On positron-emission tomography CT fusion, the uptake value of 18-fluoro-2-deoxy-D-glucose decreased markedly in the central part of the lesion, but it remained high in the peripheral part of the tumor. It was possible that the peripheral part of the lesion could not be sufficiently irradiated by SRT.

Though it was a special case, a 43-year-old man who had systemic bone metastases from FTC underwent SRT for 29 BM lesions, including a 70-mm-diameter skull lesion pressing the brain. He could not receive RAI therapy before SRT due to the risk of brain edema. One of the advantages of SRT is to be able to irradiate a large number of lesions with a small number of fractions per lesion. However, it is not common to give multiple local therapies for systemic metastatic lesions in terms of prognosis, and it is necessary to carefully consider the indications of SRT for a patient with multiple metastatic lesions.

TKI treatment is a new option for DTC patients with RAI-refractory and/or unresectable progressive disease, including bone metastases [20]. In phase 3 trials, sorafenib, vandetanib, lenvatinib and cabozantinib were shown to be effective for locally advanced or metastatic thyroid cancer, and they are currently in clinical use [21–24]. In the phase 3 study of lenvatinib in DTC, the mean percentage change from baseline in bone metastases was −10.7% for lenvatinib vs 6.5% for placebo (*P* < 0.01) [22]. However, there are several problems associated with the use of TKIs, such as complicated and cumbersome adverse events, rapid lesion regrowth following temporary interruption of TKI treatment and high treatment costs [20]. Thus, TKIs should be considered for progressive, symptomatic and/or imminently threatening DTC in which satisfactory control using RAI therapy or directed approaches (e.g. surgery, radiation therapy including SRT) is not considered likely [3, 7]. In our view, SRT appears to be a useful preliminary step to TKI treatment.

The present study has some limitations, including the short follow-up period for assessing therapeutic effectiveness and the small sample size. In addition, the retrospective design may have led to unstable results due to incomplete data and inclusion and exclusion biases.

In conclusion, based on the results of the present study, SRT using the CyberKnife system appears feasible and effective as treatment that suppresses the growth of bone metastases from DTC in selected patients.

## CONFLICT OF INTEREST

No competing financial interests exist.
